# Future climate doubles the risk of hydraulic failure in a wet tropical forest

**DOI:** 10.1111/nph.19956

**Published:** 2024-07-18

**Authors:** Zachary Robbins, Jeffrey Chambers, Rutuja Chitra‐Tarak, Bradley Christoffersen, L. Turin Dickman, Rosie Fisher, Alex Jonko, Ryan Knox, Charles Koven, Lara Kueppers, Nate McDowell, Chonggang Xu

**Affiliations:** ^1^ Earth & Environmental Sciences Division Los Alamos National Laboratory Los Alamos NM 87545 USA; ^2^ Climate and Ecosystem Sciences Division Lawrence Berkeley National Laboratory Berkeley CA 94720 USA; ^3^ School of Integrative Biological and Chemical Sciences University of Texas Rio Grande Valley Edinburg TX 78539 USA; ^4^ CICERO Center for International Climate Research Postboks 1129 Blindern 0318 Oslo Norway; ^5^ Atmospheric Sciences & Global Change Division Pacific Northwest National Laboratory Richland WA 99354 USA

**Keywords:** Barro Colorado Island, FATES, future drought, hydraulic failure, tropical forests

## Abstract

- Future climate presents conflicting implications for forest biomass. We evaluate how plant hydraulic traits, elevated CO_2_ levels, warming, and changes in precipitation affect forest primary productivity, evapotranspiration, and the risk of hydraulic failure.
- We used a dynamic vegetation model with plant hydrodynamics (FATES‐HYDRO) to simulate the stand‐level responses to future climate changes in a wet tropical forest in Barro Colorado Island, Panama. We calibrated the model by selecting plant trait assemblages that performed well against observations. These assemblages were run with temperature and precipitation changes for two greenhouse gas emission scenarios (2086–2100: SSP2‐45, SSP5‐85) and two CO_2_ levels (contemporary, anticipated).
- The risk of hydraulic failure is projected to increase from a contemporary rate of 5.7% to 10.1–11.3% under future climate scenarios, and, crucially, elevated CO_2_ provided only slight amelioration. By contrast, elevated CO_2_ mitigated GPP reductions. We attribute a greater variation in hydraulic failure risk to trait assemblages than to either CO_2_ or climate.
- Our results project forests with both faster growth (through productivity increases) and higher mortality rates (through increasing rates of hydraulic failure) in the neo‐tropics accompanied by certain trait plant assemblages becoming nonviable.

## Abstract 2

See also the Commentary on this article by Mackay, **244**: 2115–2117.

## Introduction

Tropical forests have large impacts on global water and carbon cycles. Of global terrestrial systems, they intercept *c*. 35% of precipitation, contribute *c*. 70% of transpiration and 33% of evapotranspiration, and account for 60% of photosynthesis (Beer *et al*., 2010; Schlesinger & Jasechko, 2014). The trajectories of these tropical water and carbon fluxes are uncertain due to the potential impacts of global climate change (Mitchard, 2018). A modern increase in the prevalence of droughts (since 2000) has resulted in significant declines in the Amazon and is attributed as causing a 50% decrease in normalized difference in vegetation index, with significantly more loss in drought‐impacted forests (Xu *et al*., 2011; Hilker *et al*., 2014). Further, a recent trend of increasing drought has also been shown to reduce primary productivity in tropical forests (Zhang *et al*., 2016). This is supported by recent site‐level studies which have shown that the carbon storage potential of these forests has declined in recent years due to an increase in mortality rates that may offset future gains in productivity owing to warmer temperatures, CO_2_ fertilization, and reforestation (Brienen *et al*., 2015).

Projecting of future gross primary productivity and mortality is complex, owing to the interdependent (and sometimes counteracting) effects of rising CO_2_, vapor pressure deficit (VPD; Grossiord *et al*., 2020), and changes to frequency and severity of drought (Chiang *et al*., 2021). GPP is reduced by increasing atmospheric aridity (VPD) through stomatal closure, which in turn reduces the risk of hydraulic failure (or irreversible desiccation through loss of hydraulic transport capacity; McDowell *et al*., 2022). However, hydraulic failure is exacerbated when increased atmospheric demand for water exceeds available water in the soil (Bartlett *et al*., 2012; Meng *et al*., 2022). Increased CO_2_ concentration may offset part of future drought stress by increasing water use efficiency (WUE: the amount of water loss by transpiration per unit of carbon fixed by photosynthesis; Briggs & Shantz, 1913) thus increasing GPP and reducing hydraulic failure risk (Eamus, 1991; Adams *et al*., 2017; Wu *et al*., 2020). During drought, however, base transpiration rates are often low, which may limit or counteract any benefit of these effects (Bartlett *et al*., 2012; Adams *et al*., 2017; Matheny *et al*., 2017).

Due to broad variation in hydraulic trait assemblage (i.e. properties determining plant hydraulic conductivities and vulnerability to water stress), the net impacts of rising VPD, drought, and CO_2_ on GPP and risk of hydraulic failure is unknown (Choat *et al*., 2018). Prior research has focused on lone traits under increasing aridity; however, water transport (leaf and sapwood area, conductivity rate), leaf behavior (hydraulic safety margin, stomatal closure rate) and xylem safety (hydraulic conductivity, xylem embolism resistance) are often controlled by trait assemblages and their interactions (Meinzer *et al*., 2005; Bartlett *et al*., 2012; Choat *et al*., 2012; Li *et al*., 2015; Tavares *et al*., 2023). Process‐based plant hydrodynamics modeling allows investigation of how different plant traits will affect emerging plant behaviors under future climate condition.

In this study we investigated the impacts of future climate and CO_2_ on GPP and mortality risk due to hydraulic failure in a wet tropical forest on Barro Colorado Island (BCI), Panama (Supporting Information Figs S1, S2; Table S1) using a state‐of‐the‐art model that represents plant demography and whole tree hydrodynamics (FATES‐HYDRO; Xu *et al*., 2023).We hypothesized that future warming and precipitation change would lower GPP (H1A); increase the risk of hydraulic failure (H1B); higher CO_2_ levels would ameliorate GPP declines (H2A); and risk of hydraulic failure (H2B); and plant traits would account for a greater variance than CO_2_ and climate scenario in plant hydraulic failure (H3A); GPP (H3B); and Evapotranspiration (ET; H3C). We first calibrated FATES‐HYDRO; by simulating various plant trait assemblages (Table S2) and then filtering trait assemblages against observed measurements (Table S3; Fig. S3) of soil water, runoff, evapotranspiration, and gross primary productivity under a contemporary (2003–2016) climate. We then tested these hypotheses under climate projected by 16 Earth System Models (ESMs) under Coupled Model Intercomparison Project Phase 6 (CMIP6) (Table 1) and two greenhouse gas emission scenarios (Shared Socioeconomic Pathways (SSP) 2–4.5 and 5–8.5). These models were run with static stand structure (i.e. the plant size and composition are held constant) to understand the plant responses to both contemporary and projected CO_2_ and future climate, without confounding demographic or plant functional composition feedback.

**Table 1:** Summary of the climate anomaly from each model used in this study under each emissions scenario.

Model	SSP‐2 4.5 (mean annual anomaly)		SSP‐5 8.5 (mean annual anomaly)	
	Temperature (°C)	Precip. (mm)	Temperature (°C)	Precip. (mm)
ACCESS‐ESM1‐5	8.31%	−10.63%	15.58%	−9.2%
CMCC‐CM2‐SR5	7.73%	−4.7%	15.39%	−14.85%
CMCC‐ESM2	7.9%	−7.01%	14.85%	−8.29%
CNRM‐CM6‐1	10.08%	−28.33%	20.03%	−15.93%
CanESM5	9.96%	−9.02%	21.46%	−11.66%
EC‐Earth3	7.7%	−16.99%	13.14%	44.09%
EC‐Earth3‐Veg	7.33%	−8.19%	12.72%	67.7%
IITM‐ESM	5.41%	−22.62%	10.14%	−15.21%
INM‐CM4‐8	4.24%	−2.5%	8.9%	52.8%
INM‐CM5‐0	3.55%	−6.11%	8.03%	20.93%
IPSL‐CM6A‐LR	8.84%	−15.96%	18.45%	−22.73%
MIROC6	5.61%	−7.36%	12.58%	−6.4%
NESM3	6.82%	−15.39%	14.53%	−18.26%
NorESM2‐LM	7.16%	−25.44%	14.99%	−25.44%
NorESM2‐MM	7.83%	−7.74%	16.2	−61.4
TaiESM1	11.67%	−12.15%	19.55	−13.56

Each model contains two emissions scenarios or shared socioeconomic pathways (SSP2‐4.5 and SSP5‐8.5). Percent anomalies for temperature (temperature °C) and precipitation (Precip. in mm annually). Models were accessed from the CMIP6 portal (cmip6 – Home|ESGF‐CoG).

## Materials and Methods

### Study area

Barro Colorado Island (BCI), Panama, is a *c*. 1500 ha completely forested island located at 9°10′N and 79°51′W (Fig. S1). BCI has an annual mean temperature of 26.3°C and an annual mean precipitation of 2656 mm (Paton, 2020). It commonly experiences a spring dry season (*c*. 140 mm) from December to May and a wet season from May to December (*c*. 2500 mm). The forest is classified as a moist tropical forest, and much of the island remains in primary forest. Stand structure was initialized with observed data from a 50‐ha plot on site (Condit *et al*., 2017; Fig. S2; Table S1). BCI is primary composed of moist lowland tropical forest, which has been completely protected from human disturbance in the last 70 yr (Condit *et al*., 1999). This area is incredibly species diverse with 310 species regularly recorded in census (Condit *et al*., 2019; https://datadryad.org/stash/dataset/doi:10.15146/5xcp‐0d46). In the 2010 census, of the 20 species the most prevalent by basal area (m^2^ ha^−1^), comprise only 55.2% of the total basal area (Table S1). Of all trees measured within this plot 2.55% of all censused trees were > 2 cm diameter at breast height (DBH) 2.35–3.15 and 0.5% of trees were > 5 cm DBH (Fig. S2). Maximum height of the canopy averages 33 m across subplots, with a density *c*. 400 trees ha^−1^, and 2.5–3 canopy layers (Bohlman & Pacala, 2012). Our observational estimates of evapotranspiration and gross primary productivity come from a flux tower within the watershed (Pau *et al*., 2018; Larsen *et al*., 2021). The plot location simulated in this study ranges from 120 to 160 m elevation. Throughout the watershed, gravimetric soil samples are collected, and runoff is calculated via a weir from the Conrad catchment (Kupers *et al*., 2019).

### Model description

The Functionally Assembled Terrestrial Ecosystem Simulator (FATES; Fisher *et al*., 2015; Koven *et al*., 2020) is implemented through a link to a host land surface model, either the Community Land Model (CLM; Lawrence *et al*., 2019) or in the case of our study the Energy Exascale Earth System (E3SM) land model (ELM: Golaz *et al*., 2019). FATES is an ecosystem demography model structured by plant size and succession stage (age since last disturbance). For each age‐since‐last‐disturbance ‘patch’, the model tracks many ‘cohorts’ of plant consisting of populations of plants of similar size and plant functional type. For each cohort, the model provides numerical solutions to biophysical processes, including photosynthesis, respiration, allocation of carbon, water and nutrients, growth and competition, and the likelihood of mortality based on carbon starvation, hydraulic failure, and baseline background disturbances. FATES allocates carbon by photosynthesis to various vegetative pools (leaf, stem, seed, roots, storage). The variation in plant traits which control these processes allowing for varied plant carbon–water economic strategies. Further, FATES scales leaf traits through the canopy, resulting in more representative plant strategies and responses (Koven *et al*., 2020). Our simulations were run in FATES static stand mode, which does not allow for the growth of trees, dynamic change in leaf area index, or mortality within the cohorts, in order to understand the plant physiological response to future climate and CO_2_ scenarios. The full E3SM land model (FATES and the plant hydrodynamic, HYDRO, submodule) system has a parameter set that is prohibitively large for objective calibration. To facilitate parameter fitting, we leverage results from pre‐existing studies that assessed model sensitivity to a wide range of parameters (Table S2; Koven *et al*., 2020; Xu *et al*., 2023).

An optional configuration of FATES, FATES‐HYDRO, incorporates plant hydrodynamics to simulate soil to atmosphere water flow for individual trees (Christoffersen *et al*., 2016; Xu *et al*., 2023), which for the FATES vegetation demographic model, are simulated as cohorts by size class. Explicitly simulating water flow enables the direct representation of loss of hydraulic conductivity based on tissue water content change, which allows us to estimate the risk of hydraulic failure and resulting mortality rates. The water flux is calculated based on water pressure gradients across different plant compartments (rhizosphere, absorbing roots, transporting roots, stem, and leaf; Christoffersen *et al*., 2016). The water potentials for specific tissues are calculated from relative water content based on three stages of water tissue drainage. Stage one represents the water drawn from capillary reserves when the tissue is at or above full turgor. The second stage represents the stage between full turgor and the turgor loss point when the total water potential is a function of the solute and pressure water potential (elastic cell drainage). The third stage is after the turgor loss point but above the point of residual water content, where the water potential is only a function of the solute water potential.

The stomatal conductance ($g_{s}$) is simulated through a modified Ball‐Berry equation adjusted by a plant water stress factor based on the resulting leaf water potential (Eqn 1; Xu *et al*., 2023):

(Eqn 1) $$g_{s}=\left[g_{0}+g_{1}\frac{A_{n}}{C_{s}{P_{\mathrm{atm}}}^{-1}}h_{s}\right]\beta$$

where *g*_1_ is the slope of stomatal conductance in response to environmental conditions, *g*_0_ is the cuticular or minimum conductance, *C*_s_ is CO_2_ partial pressure (Pa) of the leaf surface, *P*_atm_ is the atmospheric pressure, *h*_s_ is the leaf surface humidity and *A*_n_ is the net photosynthesis rate (μmol m^−2^ s^−1^). β is a plant water stress factor calculated based on leaf water potential as follows (Eqn 2):

(Eqn 2) $$\beta ={\left[1-{\left(\frac{\psi_{\text{leaf}}}{P_{g_{s},50}}\right)}^{a_{g_{s}}}\right]}^{-1}$$

where *ψ*_leaf_ is the leaf water potential (MPa); $P_{g_{s},50}$ is the leaf water potential (MPa) at which 50% of conductance occurs and $a_{g_{s}}$ is the stomatal vulnerability parameter. Total plant hydraulic conductance and water supply changes are calculated using the water fluxes on the compartment, the leaf : shoot ratio of the plant, its height, and its hydraulic structure. See Christoffersen *et al*. (2016) and Xu *et al*. (2023) for more information on the model structure and equations.

### Model calibration

We tested and filtered a 1000‐member parameter ensemble and retained only those trait assemblages (54) for which the corresponding model outputs were close to observational data for the BCI site. Initial parameter testing ranges for plant physiological traits were selected from the parameters defined in previous studies at BCI (Table S3; Fig. S3; Koven *et al*., 2020; Xu *et al*., 2023). Parameters not included in calibration were set as those determined as optimal (Koven *et al*., 2020). To select our trait assemblages, we constrained the initial parameter space to the observational values within their range of observational error. To do this, we sampled our total parameter ensemble using a *post hoc* Markov‐chain Monte–Carlo (MCMC) approach (Table S3). Specifically, outputs were compared against a multivariate log‐normal distribution from observed monthly ET, GPP, soil water content (SWC), and runoff (including their error estimations; Fig. S2). In this process, a parameter is selected, and its log likelihood calculated; then, a second parameter set is generated from the prior's multivariate distribution (the initial distribution of each parameter). Using Mahalanobis distance (a multivariate distance metric; Mahalanobis, 1936; De Maesschalck *et al*., 2000) the model run with the lowest distance is selected, and its log likelihood is calculated. This process is iterated in the Markov‐chain Monte–Carlo process style (drawing from the prior distribution for potential moves and accepting or rejecting them based on comparative log likelihood). The range of accepted moves (i.e. the observations and their error estimations) constrains the parameter space. Final parameter space is determined by calculating the 95% confidence interval, parameters estimate, or 95% of selected models following a 10 000 step burn in (Table S3; Fig. S3). Parameter sets that resulted in no‐growth (GPP < 200 g m^−2^ yr ^−1^) or were below a precent loss of conductivity equal to or > 80% (PLC_80_; assumed a conservative, lethal threshold for hydraulic failure; Adams *et al*., 2017) under historical climate were removed before the trait assemblage selection process. The 54 trait assemblages resulting from the calibration, were then used to simulate hydraulic failure risk and indicators of plant water stress under future climate.

### Model projection

We ran each selected trait set using a future climate projection to simulate future productivity and the risk of hydraulic failure for these tropical species. To generate continuous forcing data for the future scenarios, we used CMIP6's (O'Neill *et al*., 2016) projected temperature, precipitation, and CO_2_ changes. We downloaded model outputs on historical simulations (2000–2014) and for each emissions scenario (SSP2‐4.5 and SSP5‐8.5; 2086–2100) for the pixel containing BCI (Table 1; Fig. S4). The relative weekly change between the two time periods (hence anomaly) in temperature and precipitation between the historical simulations (2000–2014) and the end of the century projection simulations (2086–2100) was calculated by week (Fig. S4). The weekly anomaly was then applied to the contemporary climate drivers for BCI used in initial model runs (2003–2016). Higher CO_2_ levels were applied depending on the emissions scenario (SSP2‐4.5: 603 CO_2_ ppm, SSP5‐8.5: 1059 CO_2_ ppm, corresponding to year 2086 in each scenario). Additionally, to understand the effects of CO_2_ on risk of hydraulic failure, we ran each model with only the applied climate anomaly, and not higher CO_2_ levels (367 CO_2_ ppm).

### Interpreting model outputs

Each trait assemblage was run under each climate scenario, and we used these outputs to analyze the change in response to water stress. We analyzed outputs as the mean across traits assemblages for each climate model for 60% and 80% loss of hydraulic conductivity (PLC_60_, PLC_80_), GPP, and ET. We separated out trait assemblages that experienced a risk of hydraulic failure (percent of day at PLC_60_ > 0.0 across all simulations) and those that did not, in order to compare crucial drought stress indicators (percent of months where leaf water potential was below $\psi 50_{g_{s}}$, minimum dry season leaf water potential, and mean transpiration rate). Significance in these tests was determined by a Mann–Whitney *U* test with Bonferroni correction where appropriate. We additionally analyzed the relative variance explained by emission scenarios, climate models, traits, for the outputs of GPP, ET, and PLC_60_.

## Results

### Climate

Climate conditions for future FATES‐HYDRO simulations were obtained from 16 CMIP6 climate models (Table 1) under SSP2‐4.5 and SSP5‐8.5 scenarios for the years 2084–2100. Averaged across the climate model ensemble, the mean annual temperature under SSP2‐4.5 was 28.0°C, a 2.0°C increase from the historical CMIP6 simulations (Table 1; Fig. S4). The mean annual temperature under SSP5‐8.5 was 29.9°C, a 3.9°C increase from the historical measurements. The average annual precipitation under SSP2‐4.5 was 1949 mm, a reduction of 455 mm from the historical CMIP6 simulations (Fig. S4). The annual precipitation under SSP5‐8.5 was 2039 mm, a reduction of 366 mm from the historical CMIP6 simulations. Reductions in precipitation primarily occurred early and late in the year, representing the driest weeks (1–15) and wettest weeks of the year (*c*. 40–50). Of note, the pattern of change in precipitation across climate models for a given emissions scenario is not uniform (Fig. S4; Table 1).

### Ecosystem fluxes

**Fig. 1:**
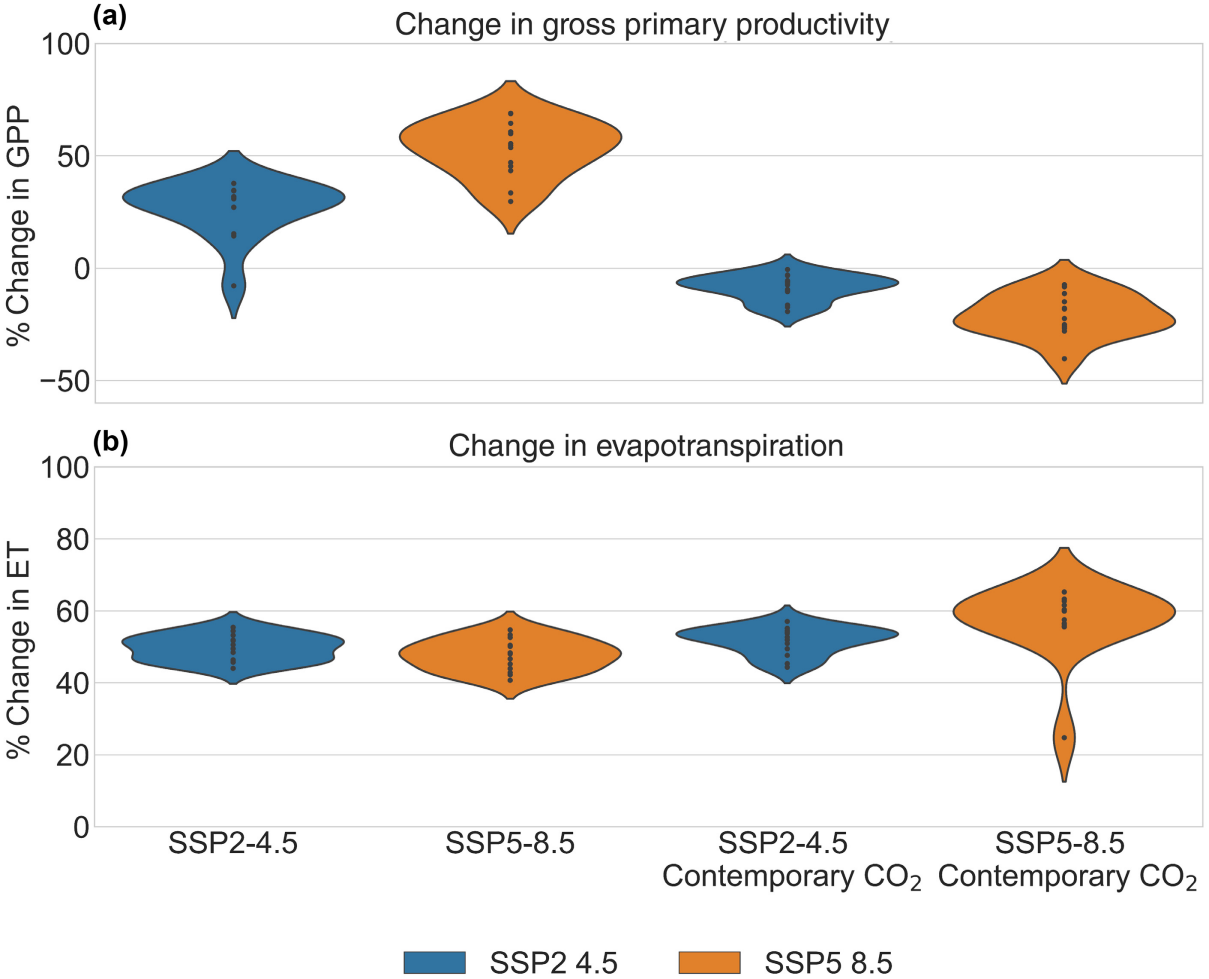
Stand‐level changes in (a) gross primary productivity (GPP); and (b) evapotranspiration (ET) projected by the FATES‐HYDRO model at Barro Colorado Island, Panama, under two climate scenarios (blue: SSP2‐4.5 and orange: SSP5‐8.5: 2086–2100) and two CO_2_ scenarios (anticipated: SSP2‐4.5 603 ppm and SSP5‐8.5 1059 ppm, and contemporary: 367 CO_2_ ppm), relative to contemporary climate (2003–2016) simulations. Each point represents the mean outcome across trait assemblages for a climate model.

To understand the impact of projected changes in climate on carbon and water fluxes, we analyzed the relative change in GPP and ET as averages across all 54 selected plant trait assemblages within a given climate model, for a given scenario. Using projected climate from the end of the century (2084–2100), SSP2‐4.5 and SSP5‐8.5 scenarios with elevated CO_2_ levels (SSP2‐4.5: 603 ppm and SSP5‐8.5: 1059 ppm) had a mean increase in GPP. SSP2‐4.5 increased by 26.7% ((−7.9%, 37.7%): representing the range of climate model uncertainty) and SSP5‐8.5 by 53.3% (29.7%, 68.9%) when compared to contemporary simulations (Fig. 1a). When CO_2_ was held at contemporary levels (367 ppm), GPP decreased by 8.6% on average (−19.3%, −0.5%) under SSP2‐4.5 and 21.0% (−40.3%, −7.4%) under SSP5‐8.5 climates. These results support our H1A hypothesis that GPP will be reduced under warmer and drier future climate and support our H2A hypothesis because GPP declines due to climate are more than compensated by elevated CO_2_.

We also examined stand‐level ET responses for their influence on ecosystem drying. Our results highlight that climate will increase the total water loss from the system, with relatively small mitigation by rising CO_2_. The SSP2‐4.5 and SSP5‐8.5 scenarios with elevated CO_2_ projected ET increases of 49.7% (44.0%, 55.4%) and 47.7% (40.7%, 54.7%), respectively (Fig. 1b). With contemporary CO_2_, the SSP2‐4.5 scenario had increases of 51.7% (44.3%, 57.1%) and SSP5‐8.5 had increases of 57.4% (24.8%, 65.3%). It should, however, be that our results assume no change in LAI and a CO_2_‐driven increase in LAI would be expected to increase transpiration further, exacerbating ecosystem drying.

### Hydraulic failure

**Fig. 2:**
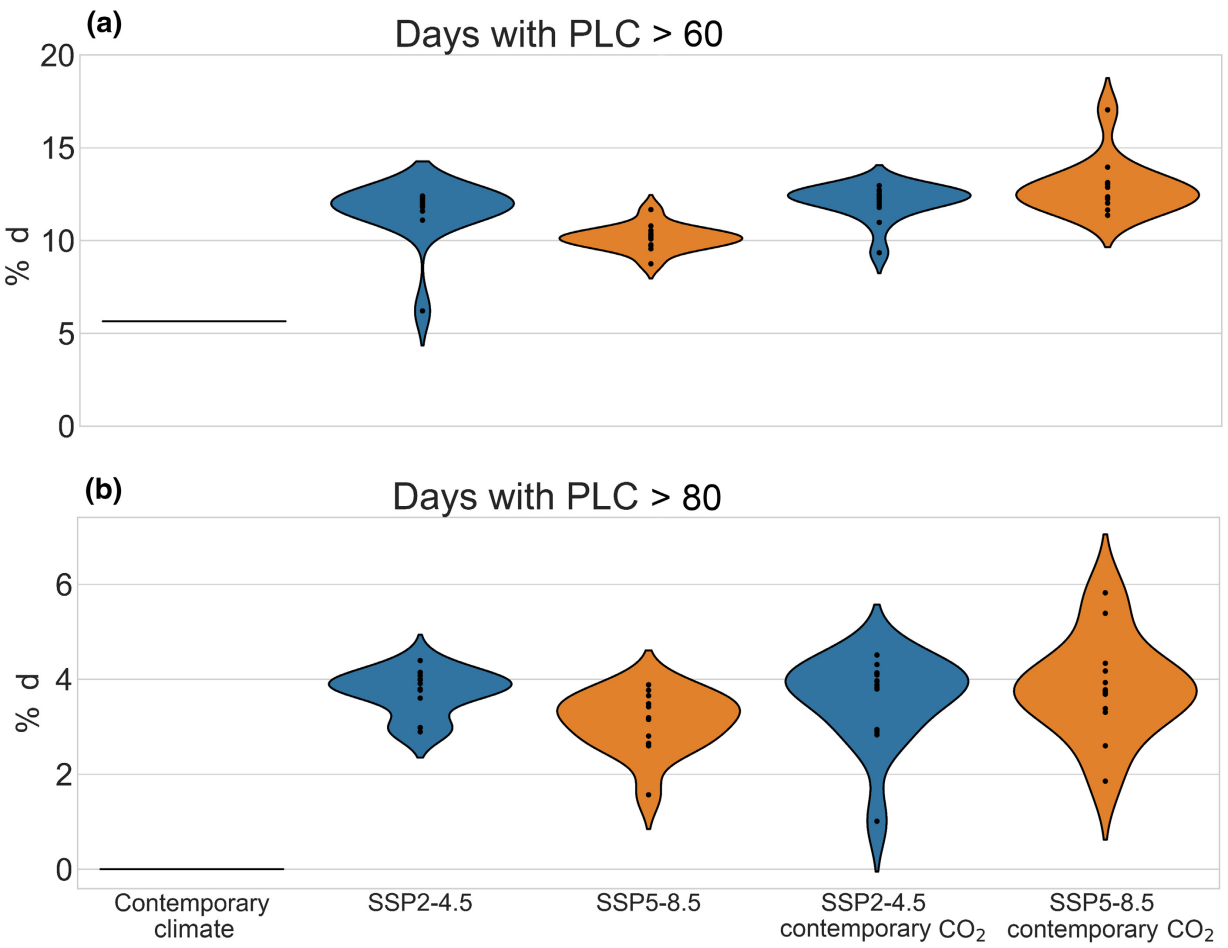
Percentage of days with (a) hydraulic failure at > 60% loss of conductivity (PLC_60_) and (b) hydraulic failure at > 80% loss of conductivity (PLC_80_), projected by the vegetation model, FATES‐HYDRO, at Barro Colorado Island, Panama, under contemporary climate conditions (2003–2016), two future climate scenarios (blue: SSP2‐4.5 and orange: SSP5‐8.5: 2086–2100) and two corresponding CO_2_ levels (anticipated: SSP2‐4.5 603 ppm and SSP5‐8.5 1059 ppm and contemporary: 367 CO_2_ ppm). Each point represents the mean outcome across trait assemblages for a climate model, thus, for the contemporary climate case, only one point represents the model run under observed conditions.

We quantified the risk of hydraulic failure as the percentage of days exceeding a percent loss of branch conductivity of 60% (PLC_60_) and 80% (PLC_80_) thresholds for risk of mortality due to hydraulic failure. We analyzed the percentage of days exceeding PLC_60_ by averaging all selected plant trait assemblages within a given climate model, for a given scenario. Under the contemporary climate, 5.7% of all days were above PLC_60_ and zero days were above PLC_80_ (Fig. 2a). Further, only two of the 54 trait assemblages experienced days where that exceeded PLC_60_. With elevated CO_2_, both SSP2‐4.5 and SSP5‐8.5 scenarios, our model predicts that 11.6% (6.2%, 12.4%) and 10.1% (8.7, 11.6%) of days would exceed PLC_60_ by the end of this century, equal to 105% and 78% increases relative to contemporary climate (Fig. 2a). With contemporary CO_2_ levels the two scenarios yielded slightly higher frequency of days exceeding PLC_60_; 12.1% (9.3%, 13.0%) under SSP2‐4.5 averaging and 12.9% (11.4%, 17.0%) under SSP5‐8.5. Similar patterns were observed when we used PLC_80_ as the cut‐off threshold for hydraulic failure, with SSP2‐4.5 and SSP5‐8.5 having increases from zero days in the contemporary climate to 3.7% (2.9%, 4.4%) and 3.1% (1.6%, 3.9%) of days with elevate CO_2_, and 3.6% (1.0%, 4.5%) and 3.8% (1.9%, 5.8%) of days without elevated CO_2_ (Fig. 2b). Our results support H1B that future warming and precipitation change will increase the future risk of hydraulic failure, but do not support our H2B hypothesis that CO_2_ will offset this mortality risk. We additionally looked at within model correlations for hydraulic failure. When averaging across trait assemblages, minimum annual soil water content explained 31% of variance in predicted failure risk under all scenarios with elevated CO_2_, while vapor pressure explained 19% of variance (Fig. S5).

### Leaf responses to climate

**Fig. 3:**
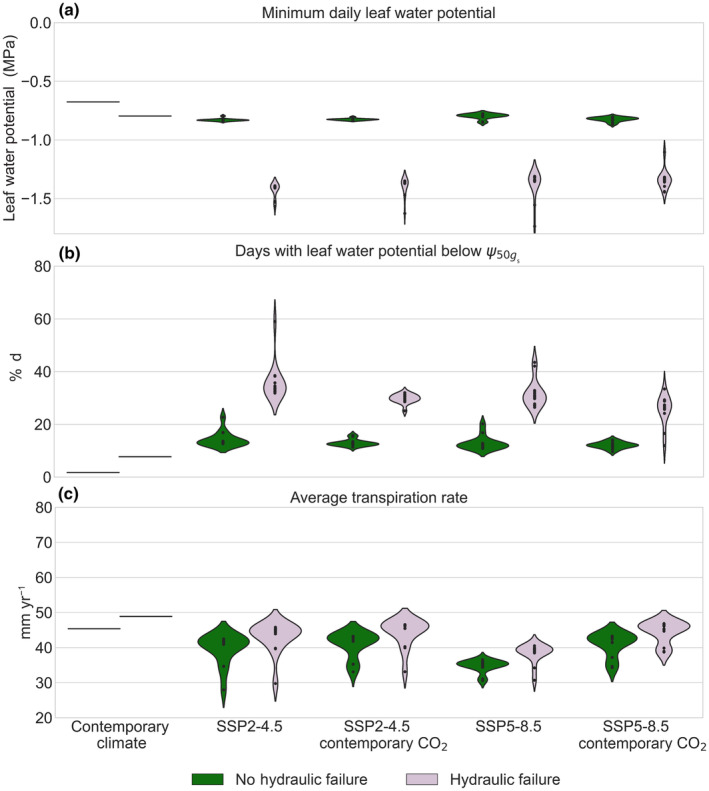
Indicators of plant stress conditioned by trait assemblage members that either experienced days with PLC_60_ in any simulation (hydraulic failure; purple) or did not (no hydraulic failure; green) as projected by FATES‐HYDRO model at Barro Colorado Island, Panama, under contemporary climate(2003–2016), the two climate scenarios (SSP2‐4.5 and SSP5‐8.5: 2086–2100) and two CO_2_ scenarios (anticipated: SSP2‐4.5 603 ppm and SSP5‐8.5 1059 ppm, and contemporary: 367 CO_2_ ppm). Plant stress indicators presented include the (a) minimum leaf water potential reached by a trait assemblage across simulations, (b) percentage of days where a trait assemblage's leaf water potential is more negative than the threshold at which stomatal conductance reaches 50% ($\psi 50_{g_{s}}$), (c) mean transpiration rate (mm yr^−1^). Each point represents the mean outcome for a climate model; thus, the contemporary climate simulation only has one point representing the model run under observed conditions (2003–2016). Note in the case of contemporary simulations, this represents species that would go on to experience hydraulic failure (left, green) or not (right, purple) under future, not contemporary, simulation.

Minimum annual leaf water potentials represent a measurement of the maximum water stress a plant is under with more negative potentials representing greater stress. Minimum annual leaf water potential under contemporary simulations was −0.735 MPa (Fig. 3a); this decreased under both future scenarios with anticipated CO_2_ (SSP2‐4.5: −1.129 MPa; SSP5‐8.5: −1.088 MPa) which was similar to contemporary CO_2_ scenarios (SSP2‐4.5 contemporary CO_2_: −1.113 MPa; SSP5‐8.5 contemporary CO_2_: −1.0816 MPa; Fig. S6). Trait assemblage members that experienced hydraulic failure had substantially more negative minimum season leaf water potential (−1.376 MPa) than those who did not (−0.815 MPa; Fig. 3a).

$\psi 50_{g_{s}}$ is the leaf water potential (a measure of water stress) at which the plant reduces its stomatal conductance by 50%. In contemporary simulations, only 1.7% of days across all trait assemblages had leaf water potentials more negative than each assemblage's respective $\psi 50_{g_{s}}$ (Fig. 3b). Under the elevated CO_2_, scenario SSP2‐4.5, the percentage of days exceeding $\psi 50_{g_{s}}$ increased to 16.3% (14.7%, 24.9%); under SSP5‐8.5, it increased to 14.7% (12.5%, 22.1%). The simulations with contemporary CO_2_ levels had leaf water potential exceed $\psi 50_{g_{s}}$ of all days simulated at 14.6% (12.7%, 17.4%) under SSP2‐4.5 and at 13.4% (0.9%, 15.9%) under SSP5‐8.5 (Figs 3b, S7). Under all scenarios, the trait assemblages that experienced hydraulic failure (PLC_60_) spent significantly greater time with leaf water potential exceeding $\psi 50_{g_{s}}$ than those that did not. Trait assemblages that experienced hydraulic failure across all future scenarios had a 136% increase in the percentage of days exceeding the $\psi 50_{g_{s}}$ threshold (i.e. 30.3% during 2084–2100 compared to 12.8% during contemporary simulations).

Mean plant transpiration (Fig. 3c) under contemporary climate (47.1 mm yr^−1^) was higher than that in future scenarios. This is likely because transpiration represents both the amount of water lost to rising vapor pressure deficit, but also the amount of time stomata remains open. Increasing days with leaf water potentials above $\psi 50_{g_{s}}$ and the resulting stomatal closure reduce the overall transpiration rate. Scenarios with higher CO_2_ levels had lower transpiration rates (SSP2‐4.5: 41.4 mm yr^−1^, SSP5‐8.5: 36.6 mm yr^−1^) than equivalent contemporary CO_2_ simulations (SSP2‐4.5 contemporary CO_2_: 42.7 mm yr^−1^; SSP5‐8.5, contemporary CO_2_: 42.8 mm yr^−1^). Trait assemblages that experienced hydraulic failure had higher transpiration rates on average (42.6 mm yr^−1^) than those that did not (39.2 mm yr^−1^), though the difference was only significant under the SSP2‐4.5 and SSP2‐4.5 scenarios with contemporary CO_2_.

### Importance of drivers for variance in hydraulic failure risk, GPP, and ET rates

**Fig. 4:**
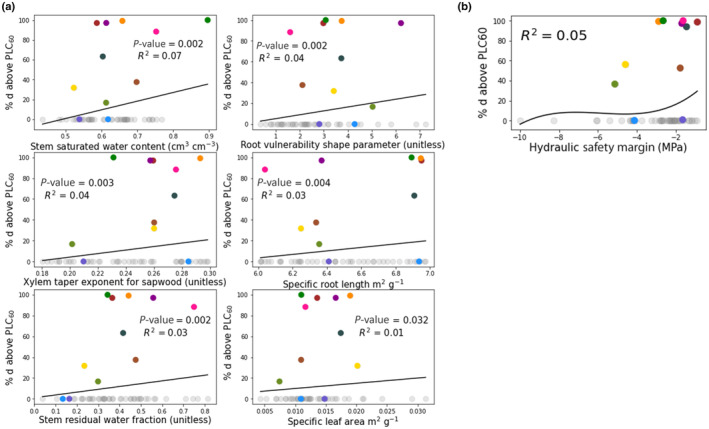
Plant trait contributions to hydraulic failure in FATES‐HYDRO simulations for Barro Colorado Island, Panama: (a) Percentage of days with > 60% loss of conductivity (PLC_60_) as a function of the six primary traits responsible for hydraulic failure in our simulations: stem saturated water content (cm^3^ cm^−3^), xylem taper exponent for sapwood (unitless), stem residual water fraction (unitless), root vulnerability shape parameter (unitless), specific root length (m g^−1^), specific leaf area (m^2^ g^−1^) and (b) the contribution of hydraulic safety margin (not a parameter but an emergent property). Only traits with significant regressor terms (*P* < 0.05) and *R* ^2^ > 0.01 are included. Each point represents a trait assemblage, gray points experienced no hydraulic failure, colored points experienced at least 1 d across all simulations with mean PLC greater than PLC_60_. Colors for trait assemblages are consistent across panels.

We examined to which degree modified traits in plant trait assemblages could explain risk of hydraulic failure. All significant traits (*P* < 0.05) had low explanatory value (*R* ^2^ ≤ 0.07). The most explanatory trait was stem saturated water content (*R* ^2^ = 0.07), followed by root vulnerability shape, xylem taper, and specific root length (Fig. 4a). No single trait dominantly explains the hydraulic vulnerability. Low explanation of variance (*R* ^2^ ≤ 0.1) and inclusion of trait assemblages with no hydraulic failure (Fig. 4a) across the individual trait spaces highlight that it is various trait combinations, particularly those that contribute to differences in minimum annual leaf water potential or days below $\psi 50_{g_{s}}$, rather than individual traits that result in projected days exceeding PLC_60_ (Figs S7, S8; Table S4). We additionally tested the property of hydraulic safety margin (the difference between the minimum xylem water potential and the P50_stem_) which can be calculated from FATES‐HYDRO but is not a parameter, this additionally explained very little variance (Fig. 4b). We further decomposed the variance across all simulations to understand the relative contribution of emissions scenarios (SSP2‐4.5 and SSP5‐8.5), CO_2_, climate model variance, and plant trait assembles regarding hydraulic failure, GPP, and ET (Table 2; H3A, H3B, and H3C). Across all simulations, traits overwhelmingly accounted for the greatest proportion of variation in hydraulic failure outcomes, with climate scenarios, climate model and CO_2_ playing a marginal role (Table 2). GPP was also primarily explained by traits, but both emissions scenario and CO_2_ played a significant role in determining GPP, while climate model variance played a minor role. For ET, traits played a primary role with climate, emissions scenario and CO_2_ playing minor roles.

**Table 2:** The relative contributions of climate models, trait ensembles, climate scenarios, and CO_2_ concentration to variance in hydrologic failure (60% percent loss of conductivity: PLC_60_), gross primary productivity, and evapotranspiration in FATES‐HYDRO simulations of Barro Colorado Island under two or shared socioeconomic pathways (SSP2‐4.5 and SSP5‐8.5) and two CO_2_ scenarios (anticipated: SSP2‐4.5 603 ppm and SSP5‐8.5 1059 ppm, and contemporary: 367 CO_2_ ppm).

Variable	Hydraulic failure (PLC_60_)	Gross primary productivity	Evapotranspiration
Climate model	< 0.1%	2.2%	1.7%
Trait ensemble	98.2%	64.4%	97.1%
Emissions scenario	< 0.1%	20.1%	0.5%
CO_2_ level	< 0.1%	27.2%	< 0.1%

Simulations were run for each trait assemblage under each climate model, and output was decomposed to calculate the percentage of variance explained.

## Discussion

Using a mechanist hydrodynamic vegetation model of the tropical forests of Barro Colorado Island, we examined the impact of climate scenarios, plant hydrodynamic traits, and increased CO_2_ levels on plant hydraulic stress, risk of hydraulic failure, and photosynthetic productivity. Driven by climate data from 16 CMIP6 climate models, FATES‐HYDRO projects that these tropical plants will increasingly experience mortality risk due to hydraulic failure under future climates. Our results suggest a doubling of hydraulic failure under SSP2‐4.5 and a near doubling under SSP5‐8.5 with a relatively small but significant variance when accounting for increasing CO_2_ levels. Like previous work, we see that no one plant trait completely determines the risk of hydraulic failure in FATES‐HYDRO; instead, it is the combination of traits that determines the risk of hydraulic failure under future climate change (Fig. 4; Xu *et al*., 2023).

Our research shows the likelihood of hydraulic failure under future climate hinges on understanding the interaction of plant trait assemblages. In comparing the relative contributions of climate models and greenhouse gas emission scenarios, CO_2_, and plant hydraulic traits to the likelihood of hydraulic failure, plant hydraulic traits are the most critical predictor of future risk of hydraulic failure. We additionally find contemporary trait assemblages which operate at similar rates of transpiration, minimum leaf water potential, and not at risk of hydraulic failure become unviable under warmer temperatures of the future (Fig. 3). This supports the theory that trait assemblages are operating at the edge of their hydraulic thresholds for mortality (Peters *et al*., 2021). However, our work suggests that plant traits may account for a far greater variance that emissions scenario in the rates of growth and mortality, ultimately introducing a high degree of uncertainty in the carbon budget due to the nonlinear response of certain forests to increased CO_2_ and changing climate. While we find traits more significant in determining the risk of hydraulic failure rates, we do see the expected relationship between decreasing soil moisture, increasing vapor pressure deficit and rising hydraulic failure (Fig. S5). As seen in other tropical forests, soil water moisture may be the dominant factor in determining changes in plant sapflow (Meng *et al*., 2022). We see that the future emissions scenarios had a negligible effect on the likelihood of hydraulic failure in the aggregate (Table S2). The change in underlying mortality resulting from increased risks of hydraulic failure can profoundly affect carbon storage and forest demographics. Large changes in aboveground biomass have been reported in areas where soil water storage is likely lower (Hasper *et al*., 2017; Tavares *et al*., 2023). If realized, a doubling of the mortality rate will reduce carbon storage by half over 50 yr in the absence of other changes in ecosystem productivity (Swann, 2018).

Our results further demonstrate the role of increased CO_2_ in maintaining productivity under increased temperature and water stress (Fig. 1). Our simulations show greater increases in GPP under scenarios where CO_2_ enrichment is included, suggesting less water limitation in these scenarios, leading to greater overall productivity. Further, while traits still played the dominant role in determining the variance in productivity (GPP), CO_2_ and climate scenarios accounted for a substantial portion as well. Free air carbon enrichment experiments in temperate forests show an increase in water use efficiency, though not a significant decrease in transpiration, instead resulting in increased productivity, similar to our results (Norby *et al*., 2010; Zaehle *et al*., 2014). Short term increases in CO_2_ have been shown to reduce stomatal conductance, thus increasing the water use efficiency (Cai *et al*., 2021). Distributed observations of tropical forest demographics in Amazonia and Africa have estimated an increase in the total amount of biomass (*c*. 30%) in recent decades (Swann, 2018). Within the context of FATES‐HYDRO, stomatal closure and transpiration is calculated by a Ball‐Berry stomatal conductance scheme, which governs the exchange of CO_2_ necessary to maintain photosynthesis (Xu *et al*., 2023). Overall, from our results, the projected increases in atmospheric water demand are projected to overwhelm the buffering effects of CO_2_ in terms of hydraulic failure, likely leading to faster growth, faster mortality in tropical forest, with unclear net effects on carbon storage. It is additionally unclear how nutrient limitations will impact plant carbon budgets in a CO_2_ rich future (Norby *et al*., 2010; Zaehle *et al*., 2014). Plants may expend greater resources on nutrient acquisition, thereby offsetting increases in GPP. Future experiments with FATES‐HYDRO will seek to evaluate hydraulic and nutrient limitations simultaneously in efforts to incorporate nutrient acquisition costs into constraining plant traits.

Reduction in viable trait assemblages under future climate and CO_2_ may serve as a functional trait filter (through increased mortality), reorienting the trait composition toward assemblages able to take advantage of higher productivity while maintaining hydraulic safety margins and lower leaf water potentials (Gallagher *et al*., 2013; Powell *et al*., 2018; Tavares *et al*., 2023). Additionally higher tree mortality rates may increase gaps in the canopy, favoring short‐lived pioneer species establishment (Kumagai & Porporato, 2012) and possibly compounding mortality in the long‐term (Swann, 2018). High risk of hydraulic failure was, in part, associated with trait assemblages with lower hydraulic safety margins (Fig. 4b). Plant hydraulic safety margins are considered narrow regardless of the biome, and our results support that assertation that plants operate within a range near their hydraulic failure (Choat *et al*., 2012). Under contemporary climate, trait assemblages that would experience hydraulic failure under future climate scenarios showed low variations in minimum leaf water potential and months exceeding $\psi 50_{g_{s}}$. Our simulations indicate that, even under warmer or drier scenarios, certain species can modulate their hydraulic dynamics (by, for example closing their stomata can maintain less negative stem water potentials) and avoid hydraulic failure (Fig. 4). As shown in other studies of the tropics, species at BCI that cannot modulate their leaf water potentials will be more vulnerable to hydraulic failure (Aguirre‐Gutiérrez *et al*., 2020). Similarly, observations and modeling have shown a shift in the last three decades toward more xeric species across the tropics in conditions where water is limited (Yang *et al*., 2015; Sakschewski *et al*., 2016).

The role of increased water uses efficiency (via CO_2_ enrichment), increasing evaporative demand (via rising temperature) and change in the transpiration rate of plants (via multiple factors) can all combine to affect future soil water moisture and water residence time (Zhang *et al*., 2016). We see here even as transpiration rate remains constant, rising evaporative rate can increase total evapotranspiration considerably. Under all scenarios we see a considerable increase in the evapotranspiration (*c*. 20–40%) of these tropical forests (Fig. 1b). This increase in water removed from the forests, coupled with decreasing precipitation rates in future climate scenarios results in less water available for plant maintenance and is likely a partially a driver of increased risk of hydraulic failure. In our simulations transpiration rates were reduced or stable due to more negative leaf water potentials and thus increased duration of stomatal closure (Fig. 4). Bonal *et al*. (2016) assessed the contribution of plant transpiration and evaporation to total evapotranspiration and saw rising ET levels, however, this was primarily attributed to increased transpiration in plants in the Americas. It should be noted that they predict a net decrease in ET due to decreasing soil evaporation and rise in gross transpiration both associated with increased LAI coverage, which was not simulated in our static stand mode simulations. Our results also contrast with similar reanalysis studies of the Eurasian continent suggesting that increases in evapotranspiration due to recent increases in temperature and leaf area may be offset by increased water use efficiency (Zhang *et al*., 2021).

The use of FATES‐HYDRO to address the risk of hydraulic failure represents a major breakthrough in modeling hydraulic failure in tropical species. The Penman‐Monteith‐Leuning (PML) model only uses the maximum stomatal conductance, the photosynthetically active radiation, and the radiation and water vapor deficit ratios when half of the maximum stomatal conductance is reduced. These models have shown evapotranspiration in tropical regions to be highly transpiration‐dependent due to energy limitations in evaporative demand (Zhang *et al*., 2016). However, these models assume a static relationship between the stress on the plant (as enforced by water deficits) and do not account for the way increasing CO_2_ and vapor pressure deficit may provide nonlinear effects on transpiration. In simulations of separate tropical forests (Powell *et al*., 2013), an ensemble of land surface models (IBIS, JULES, SIB3, ED2, CLM 3.5) that use soil matrix potential or soil water content as a regulator of carbon assimilation and evapotranspiration showed a *c*. 50 to 100% reduction in the gross primary productivity under simulations with a 50% precipitation reduction (a far more significant reduction, than that projected in the climate projection). These models capture empirical changes in the stomatal conductance rather than the leaf water potentials and do not consider the multiple methods of hydraulic maintenance. In the past decade, there has been a proliferation of development of plant hydrodynamic models for Earth System Models to better capture plant response to droughts. These models can be divided into PFT‐dependent big‐leaf models (e.g. LPJ: Hickler *et al*., 2006; CLM5‐PHS: Kennedy *et al*., 2019; CABLE‐Desica: De Kauwe *et al*., 2020; Noah‐MP‐PHS: Li *et al*., 2021 and JULES‐SOX: Eller *et al*., 2018), and size and PFT‐dependent ecosystem demography models (e.g. ED2: Xu *et al*., 2016 and FATES‐HYDRO: Xu *et al*., 2023). While these models showed a better prediction of vegetation response to droughts compared to the default nonhydrodynamic versions, so far there are limited studies that assess the future climate impact on hydraulic failure. Our results compare with those of Powell *et al*. (2018) who found that aboveground biomass simulated for the BCI site remained similar in the future under all except the driest of conditions, primarily due to an adjustment in demography favoring more drought‐resistant species and increased regeneration in the immediate years following mortality. Powell *et al*. used ED2‐Hydro, which simulates water stress within plants using empirical relationships which account for xylem stress and stomatal closure. While Powell *et al*. ran simulations under heuristic drought scenarios, we simulated climate from 16 CMIP6 climate models and multiple scenarios to capture the range of variation in future climate predictions. We additionally provided the model with enhanced levels of CO_2_ to understand its effect. Our result of no substantial CO_2_ impacts on lowering hydraulic failure risk is also different from Liu *et al*. (2017), which showed that increasing atmospheric humidity and CO_2_ concentration substantially alleviates the mortality risk based on hydraulic failure. This is likely because Liu *et al*. consider the mortality risk based on a combination of factors including loss of conductance in the xylem and the days of stomata closure (determined by stomatal conductance as a function of CO_2_), while we only consider the risk based on a mechanistic loss of conductance in xylem.

Our study should be considered within the context of the experimental design. Changes in GPP and ET should be considered only a baseline of future stand dynamics owing to the lack of adaptive cohort mortality in the simulations. While plants may have a greater productive ability, this may be offset by mortality (Li *et al*., 2015; Hasper *et al*., 2017; Tavares *et al*., 2023). To sample a wide range of future traits, our simulations are representations of an assemblage of mean trait value of a given forest trait composition. This may exaggerate effects of certain plant behaviors. We additionally recognize our study may represent one where overall projected change in VPD are small (as modeled by CMIP6), our findings may not hold in tropical forests where VPD changes are projected to be more extreme. We would additionally highlight the uncertainty in future projections of precipitation patterns, which can vary widely among CMIP models for the same future emission scenario (Table 1). In this study, we considered a wide range of tropical hydrological traits, ecologically filtered by observational values and viability, to understand how future climate may affect growth and how hydraulic failure may affect tropical forests. Our simulations were run in a static mode, which may limit their inference for long‐term trends within the tropics, as demographic processes will respond to increased mortality and decreased water availability. Further, each result represents a homogenous trait set, and diverse traits may show greater resilience to future climate. Future FATES‐HYDRO studies will use competitive plant functional type construction and demographics to study hydraulic failure in various tropical locations. However, given the diversity of many neotropical sites, it may always be necessary to represent simplified functional types, which fall short of true hydraulic trait diversity.

Similar to other studies, our results showed that climate model variance can play a larger role in simulations than emissions scenario in determining key factors in the global carbon balance (Lovenduski & Bonan, 2017). Additionally, the long‐term effect of CO_2_ enrichment on plants of the tropics is still largely a matter of debate, as most of the free air carbon enrichment and free air temperature enrichment studies have been conducted in temperate and boreal forests. For example, acclimation of photosynthetic rates to temperature, seems to vary widely by species and location, possibly reducing gains in productivity (Pau *et al*., 2018). Further, spatial heterogeneity (e.g. soils, slope, and drainage) has been shown to play a large role in mortality patterns during drought and must be considered within the context of these findings and the effects of plant traits while our model simulates and based on mean surface conditions (Li *et al*., 2015; Hasper *et al*., 2017).

Our results highlight the need for greater study of trait assemblies in the context of climate CO_2_ on net ecosystem productivity. Our results project forests with both faster growing (through productivity increases) and higher mortality rates (through increasing rates of hydraulic failure) in the neo‐tropics accompanied by certain trait plant assemblages becoming nonviable. Nonviability of existing trait assemblages will have impacts for biodiversity, as well as biogeochemical cycling. Further, our results suggest a more rapid hydrologic cycle in tropical forests, which, given the tropics' importance, has significant implications for the global water cycling. Nevertheless, understanding this large‐scale feedback will require intensive trait simulation at sites throughout the tropics to understand variability in their response. Our future research will incorporate nutrient competition and investigate how shifts in species composition will affect the net carbon and water balance across a greater number of tropical forests.

## Competing interests

None declared.

## Author contributions

ZR, CX, BC and AJ designed the research. ZR and CX performed the research. ZR, JC, CX, BC, AJ, RC‐T, LTD, RF, RK, CK, LK and NM analyzed and interpreted the data. ZR, CX, JC, BC, AJ, RC‐T, LTD, RF, RK, CK, LK and NM contributed to writing the manuscript.

## Supporting information


**Fig. S1** Map of the study area.
**Fig. S2** Distribution of plant diameters (cm) in the 50 ha plot at Barro Colorado Island, Panama, in the 2010 census (from data in Condit *et al*., 2017).
**Fig. S3** Comparison of observations (black) and 54 FATES‐HYDRO trait assemblage members for Barro Colorado Island (red) selected through a MCMC parameterization process.
**Fig. S4** Weekly ensemble means from the 16 climate models for SSP2‐4.5 (blue) and SSP5‐8.5 (orange) scenarios and Barro Colorado Island (BCI).
**Fig. S5** Correlations between 60% percent loss of conductivity (PLC_60_) and minimum annual soil water content and vapor pressure deficit.
**Fig. S6** Change in Dry season (mid‐December–early May) minimum midday leaf water potential (MPa) for the two future climate scenarios.
**Fig. S7** Comparison of climate scenarios months with leaf water potential (LWP) below $\psi 50_{g_{s}}$.
**Fig. S8** Traits as a function of risk of hydraulic failure (% months above PLC_60_) from FATES‐HYDRO model outputs averaged across SSP2‐4.5 and SSP5‐8.5 scenarios.
**Fig. S9** Additional traits as a function of risk of hydraulic failure (% months above PLC_60_) from FATES‐HYDRO model outputs averaged across SSP2‐4.5 and SSP5‐8.5 scenarios.
**Table S1** Species by percentage of the total basal area for the 50 ha plot at Barro Colorado Island, Panama, as determined in the 2010 census (from data in Condit *et al*., 2017).
**Table S2** Parameter ranges used in sample trait assemblages for BCI FATES‐HYDRO simulation.
**Table S3** Results from the sampling space search conducted by the Markov‐chain Monte–Carlo process.
**Table S4** Ten most highly correlated variables with PLC_60_ from future anomaly CMIP6 simulations of the FATES‐HYDRO model for Barro Colorado Island.
Please note: Wiley is not responsible for the content or functionality of any Supporting Information supplied by the authors. Any queries (other than missing material) should be directed to the *New Phytologist* Central Office.

## Data Availability

Results from model runs are stored in the NGEE‐tropics data archive https://ngt‐data.lbl.gov/dois/ and with ESS‐DIVE https://ess‐dive.lbl.gov/. The FATES model is available at https://github.com/NGEET/fates and the E3SM model at https://github.com/E3SM‐Project/E3SM.
