# Supplementary material for: Future climate doubles the risk of hydraulic failure in a wet tropical forest
Source: New Phytol. 2024 Jul 18;244(6):2239–50. doi: 10.1111/nph.19956 (PMC11579428; doi:10.1111/nph.19956)
Supplement: Supplementary file 1 — Fig. S1 Map of the study area. Fig. S2 Distribution of plant diameters (cm) in the 50 ha plot at Barro Colorado Island, Panama, in the 2010 census (from data in Condit et al., 2017). Fig. S3 Comparison of observations (black) and 54 FATES‐HYDRO trait assemblage members for Barro Colorado Island (red) selected through a MCMC parameterization process. Fig. S4 Weekly ensemble means from the 16 climate models for SSP2‐4.5 (blue) and SSP5‐8.5 (orange) scenarios and Barro Colorado Island (BCI). Fig. S5 Correlations between 60% percent loss of conductivity (PLC60) and minimum annual soil water content and vapor pressure deficit. Fig. S6 Change in Dry season (mid‐December–early May) minimum midday leaf water potential (MPa) for the two future climate scenarios. Fig. S7 Comparison of climate scenarios months with leaf water potential (LWP) below ψ50gs. Fig. S8 Traits as a function of risk of hydraulic failure (% months above PLC60) from FATES‐HYDRO model outputs averaged across SSP2‐4.5 and SSP5‐8.5 scenarios. Fig. S9 Additional traits as a function of risk of hydraulic failure (% months above PLC60) from FATES‐HYDRO model outputs averaged across SSP2‐4.5 and SSP5‐8.5 scenarios. Table S1 Species by percentage of the total basal area for the 50 ha plot at Barro Colorado Island, Panama, as determined in the 2010 census (from data in Condit et al., 2017). Table S2 Parameter ranges used in sample trait assemblages for BCI FATES‐HYDRO simulation. Table S3 Results from the sampling space search conducted by the Markov‐chain Monte–Carlo process. Table S4 Ten most highly correlated variables with PLC60 from future anomaly CMIP6 simulations of the FATES‐HYDRO model for Barro Colorado Island. Please note: Wiley is not responsible for the content or functionality of any Supporting Information supplied by the authors. Any queries (other than missing material) should be directed to the New Phytologist Central Office. [file NPH-244-2239-s001.docx]

New Phytologist Supporting Information

Article title: “Future climate doubles the risk of hydraulic failure in a wet tropical forest.”

Authors: Zachary Robbins, Jeffrey Chambers, Rutuja Chitra-Tarak, Bradley Christoffersen, Turin Dickman, Rosie Fisher, Alex Jonko, Ryan Knox, Charles Koven, Lara Kueppers, Nate McDowell, Chonggang Xu.

Article acceptance date: 20 June 2024

Correspondence: Zachary Robbins zjrobbins@lanl.gov **Table of contents****Figure S.1:** Map of the study area ………………………………………………………………..**1
Figure S.2:**  Distribution of plant diameters (cm) in the 50 ha plot at Barro Colorado Island, Panama, in the 2010 census (from data in Condit et al., 2017) …………………………………..**2
Table S.1** Species by percentage of the total basal area for the 50 ha plot at Barro Colorado Island, Panama, as determined in the 2010 census (from data in Condit et al., 2017)……………**3
Table S.2:** Parameter ranges used in sample trait assemblages for BCI FATES-HYDRO simulation………………………………………………………………………………………….**4**
**Table S.3:** Results from the sampling space search conducted by the Markov-chain Monte-Carlo process…………………………………………………………………………………………….**.6
Figure S.3**: Comparison of observations (black) and 54 FATES-HYDRO trait assemblage members for Barro Colorado Island (red) selected through a MCMC parameterization process…………………………………………………………………………………………….**8
Figure S.4:** Weekly ensemble means from the 16 climate models for SSP2-4.5 (blue) and SSP5-8.5 (orange) scenarios and Barro Colorado Island (BCI)…………………………………………**9
Figure S.5:** Correlations between 60% percent loss of conductivity (PLC_60_) and minimum annual soil water content and vapor pressure deficit………………………………………….....**10**
**Figure S.6:** Change in Dry-season (mid-December- early May) minimum midday leaf water potential (MPa) for the two future climate scenarios …………………………………………....**11**
**Figure S.7:** Comparison of climate scenarios Months with leaf water potential (LWP) below ψ50_gs_ …………………………………………………………………………………….……….**12**
**Figure S.8:** Traits as a function of risk of hydraulic failure (% months above PLC_60_) from FATES-HYDRO model outputs averaged across SSP2-4.5 and SSP5-8.5 scenarios……………………………………………………………..…………………………..**13**
**Figure S.9:** Additional traits as a function of risk of hydraulic failure (% months above PLC_60_) from FATES-HYDRO model outputs averaged across SSP2-4.5 and SSP5-8.5 scenarios………………………………………………………………………………………….**14
Table S.4:** 10 most highly correlated variables with PLC_60_ from future anomaly CMIP6 simulations of the FATES-HYDRO model for Barro Colorado Island……………………….…**15
References**…………………………………………………………………………………….…**16**


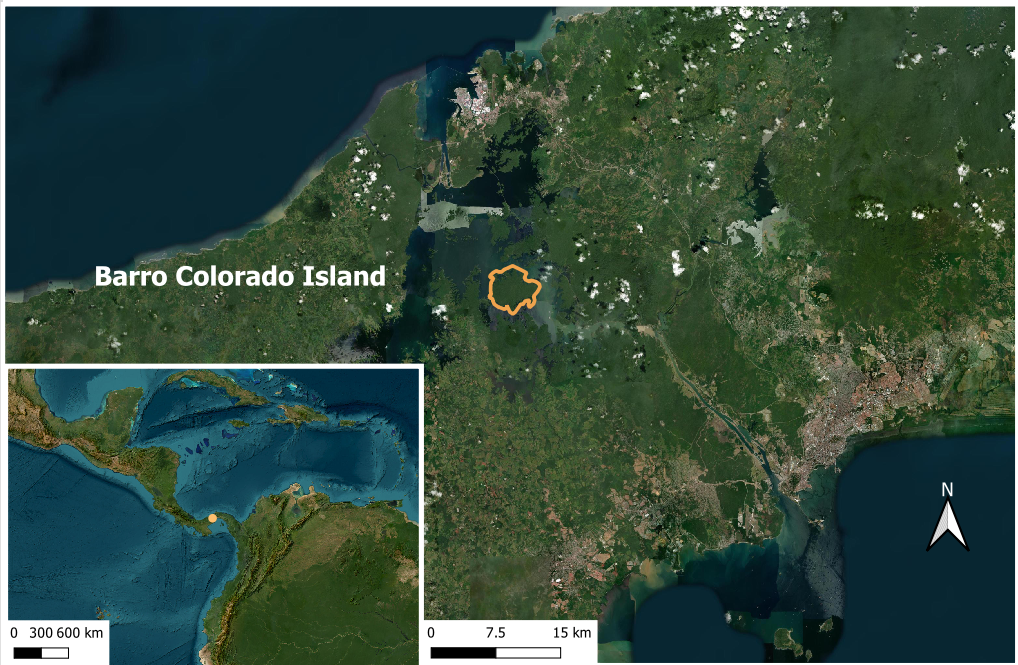
**Figure S.1:** Map of the study area, Barro Colorado Island, Panama; a ~1,500 ha completely forested island.

**
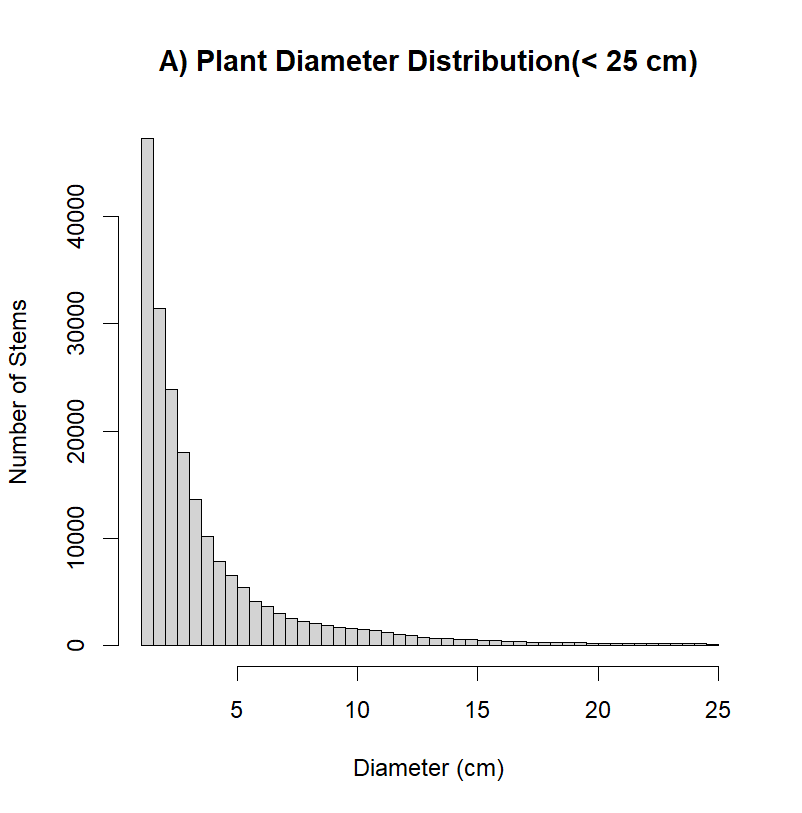
**

**
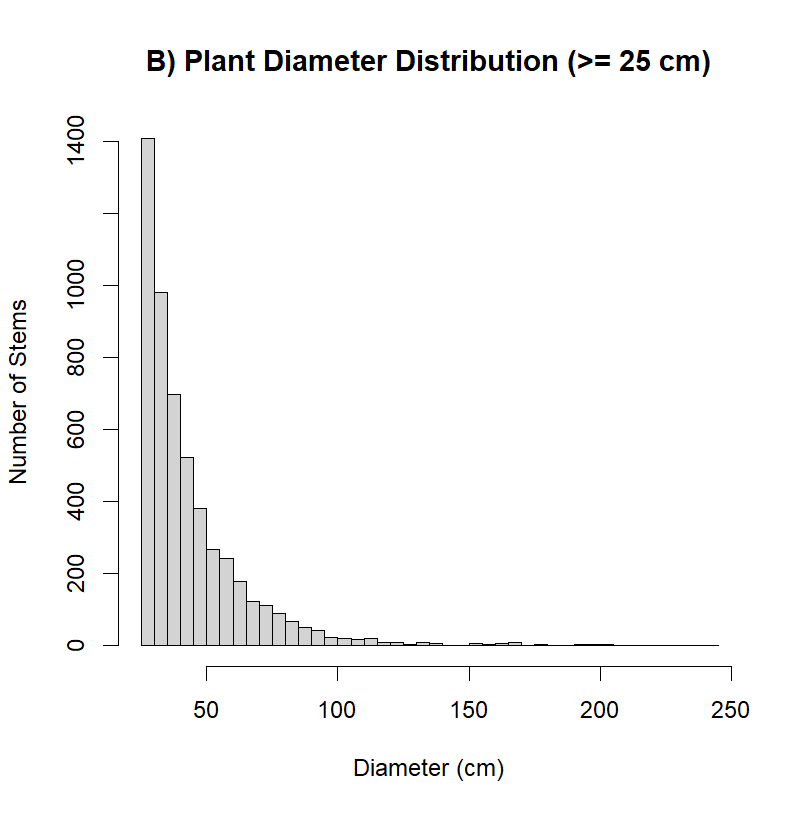
**

**Figure S.2:** Distribution of plant diameters (cm) in the 50-ha plot at Barro Colorado Island, Panama, in the 2010 census (from Condit et al., 2017); A) Diameter distribution of plants with diameter less than 25 cm, B) Diameter distribution with plants equal to or greater than 25 cm. These size distributions create the initial simulation conditions for the FATES-HYDRO Barro Colorado Island simulations in this study.

**Table S.1:** Species by percentage of the total basal area for the 50 ha plot at Barro Colorado Island, Panama, as determined in the 2010 census (from data in Condit et al., 2017). We only included species that comprised > 1 % of the total basal area in this chart, and these species only account for ~55% of the total basal area calculated within the stand. According to the census,>300 species were identified in 2010.

| ***Species*** | ***Percentage of Total Basal Area*** |
| --- | --- |
| *Quararibea asterolepis* | ***5.61%*** |
| *Prioria copaifera* | ***4.80 %*** |
| *Alseis blackiana* | ***4.70%*** |
| *Trichilia tuberculata* | ***4.39%*** |
| *Faramea occidentalis* | ***4.21%*** |
| *Hura crepitans* | ***3.98%*** |
| *Ceiba pentandra* | ***3.71%*** |
| *Oenocarpus mapora* | ***3.05 %*** |
| *Jacaranda copaia* | ***2.72%*** |
| *Anacardium excelsum* | ***1.94%*** |
| *Protium stevensonii* | ***1.90%*** |
| *Tabernaemontana arborea* | ***1.90%*** |
| *Cavanillesia platanifolia* | ***1.88%*** |
| *Apeiba membranacea* | ***1.87%*** |
| *Beilschmiedia tovarensis* | ***1.75%*** |
| *Brosimum alicastrum* | ***1.63%*** |
| *Hirtella triandra* | ***1.61%*** |
| *Ocotea whitei* | ***1.39%*** |
| *Pouteria reticulata* | ***1.32%*** |
| *Gustavia superba* | ***1.32%*** |
|  |  |
| All species listed | ***55.17%*** |

**Model parameters**
 We ran an ensemble of 1,000 (Table S.1) simulations, which differed in values for plant traits (parameters) and used a Markov Chain Monte Carlo selection process to obtain 54 parameter combinations (trait assemblages) which matched observational data (Table S.2). This model selection process compared model outputs to observations of evapotranspiration (ET), GPP, runoff, and soil water content at three depths (10, 40, and 100 cm). Compared to the observational data, the maximum RMSE of ET was 44.38 mm month^-1^, of GPP was 190.98 g C month^-1^, of runoff was 4.60 mm day^-1^, and of soil water content (SWC) at 10 cm was 0.198 mm day^-1^, at 40 cm was 0.204 mm day^-1^, and at 100 cm was 0.183 mm day^-1^ (Fig. S3).

**Table S.2:** Parameter ranges used in sampling of trait assemblages for FATES-HYDRO Barro Colorado Island, Panama, simulation. These were derived from Xu et al., 2023 and Koven et al., 2019. Model parameter abbreviation in parenthesis. U: Uniform distribution, LN: lognormal distribution, G: Gamma distribution, Beta: Beta distribution, B: Binomial distribution. Parameters were sampled and tested against observation data from Barro Colorado Island. Some parameters with the FATES-HYDRO model have unique values for each plant compartment (leaf, stem, transporting root (T_root_), absorbing root (A_root_) and are noted within the organ and range column. In the Organ column, “All” indicates the parameter is unique to each plant compartment, “Whole Plant” indicates that single value is used for each plant. Please see Xu et al., 2023 for more information on the FATES-HYDRO model.

| **Parameter (abbreviation)** | **Organ** | **Unit** | **Range** |
| --- | --- | --- | --- |
| Saturated Water Content (thetas_node) | All | cm^3^ cm^-3^ | Leaf: Beta (9.69, 6.20)  Stem, TRoot, ARoot Beta (22.98, 5.29) |
| Osmotic potential at full turgor  (pinot_node) | All | MPa | Leaf: G [9.8,6.26], Stem, TRoot, ARoot: LN [0.32,0.39] |
| Bulk elastic modulus (epsil_node) | All | MPa | Leaf: G (4.07, 4.12)  Stem, TRoot and ARoot:  G [3.57, 3.84] |
| Residual water fraction  (resid_node) | All | unitless | Leaf: B [2.14,4.10]  Stem, TRoot and ARoot:  B [2.71, 4.53] |
| Fraction of water in capillary reserve  (fcap_node) | All | unitless | U [0.1, 0.7] |
| Water potential at 50% loss of conductivity  (p50_node) | Stem and Roots | MPa | Stem, TRoot and ARoot:  G [2.07, 1.18] |
| Vulnerability curve shape  (avuln_node) | Stem and Roots | unitless | Stem, TRoot and ARoot:  LN [0.82, 0.66]. |
| Xylem conductivity per unit sapwood area  (kmax_node) | Whole plant | kg m^-1^ s^-1^ MPa^-1^ | G [1.41,2.37] |
| Xylem taper exponent for sapwood (p_taper) | Whole plant | unitless | U (0.08, 0.5) |
| Leaf area to sapwood area ratio (la2sa) | Whole plant | unitless | LN (-0.48, 0.77) |
| specific root length (srl) | Whole plant | m g^-1^ | G [1.70,35.31] |
| absorbing root radius (rs2) | Whole plant | mm | LN [1.91,0.79] |
| fraction of total tree resistance that is aboveground (rfrac_stem) | Whole plant | Unitless | U [0.1,0.7] |
| root-soil interface conductivity per unit surface area (Kr1) | Whole plant | kg m^-1^ s^-1^ MPa^-1^ | G [1.41,2.37] |
| maximum root water loss rate (K*r2)* | Whole plant | kg m^-1^ s^-1^ MPa^-1^ | LN [-6.80, 0.92] |
| Specific leaf area at top of canopy (SLA) | Whole plant | m^2^ gC^-1^ | U [0.003-0.046] |
| maximum carboxylation rate of Rub. at 25C, canopy top (Vcmax25) | Whole plant | umol CO^2^ m^-2^s^-1^ | U [30,100] |
| mean density of woody tissue in plant  (wood_density) | Whole plant | g cm^-3^ | U [0.2,0.8] |
| stomatal slope parameter, as per Ball-Berry  (ball_berry_slope) | Whole plant | unitless | U [4,16] |

**MCMC sampling**

Table S.3: Results from the parameter trait assemblage search conducted by the Markov-chain Monte-Carlo process for the FATES-HYDRO model of Barro Colorado Island, Panama. This includes the complete searched sampling space for parameters (red lines represent the 95% confidence interval, yellow lines represent the entire search space), the posterior distribution of trait values, and the autocorrelation in the sampling (horizontal line represents 0.1 autocorrelation). Markov-Chain tuning was done until autocorrelation reduced to 0.1 in under the 100 samples, and between 5-20% of samples were accepted.

| Sampling space | Posterior distribution | Autocorrelation in sampling |
| --- | --- | --- |
| 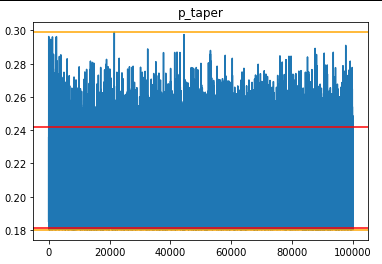 | 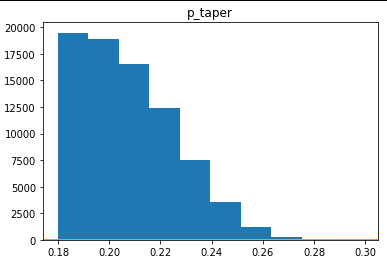 | 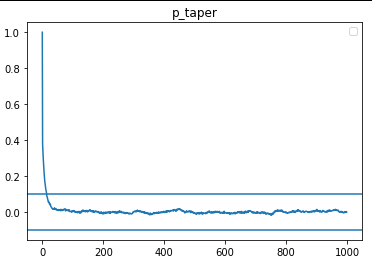 |
| 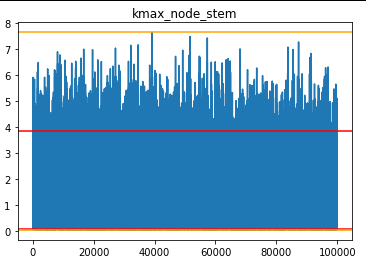 | 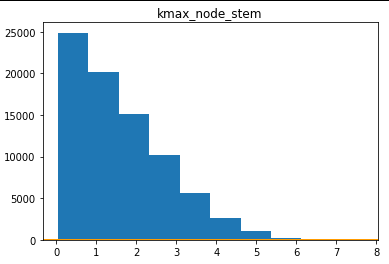 | 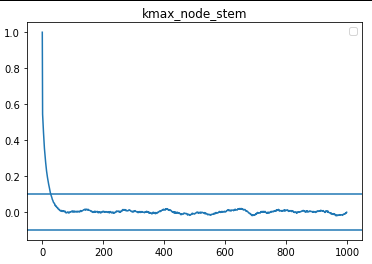 |
| 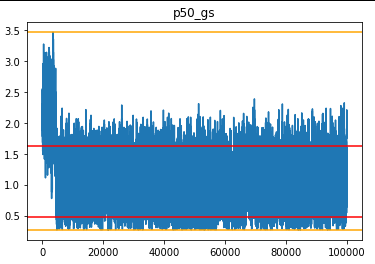 | 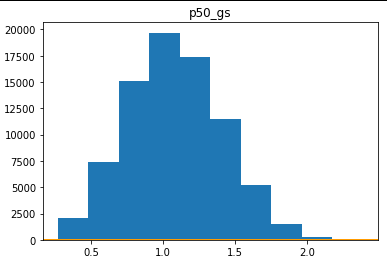 | 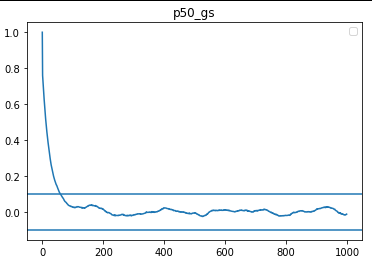 |
| 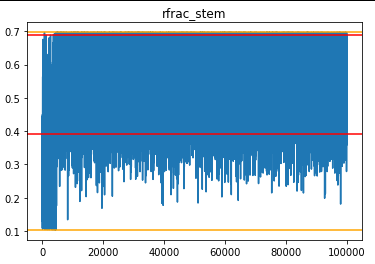 | 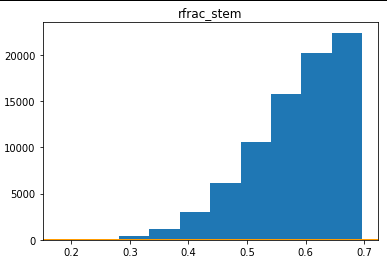 | 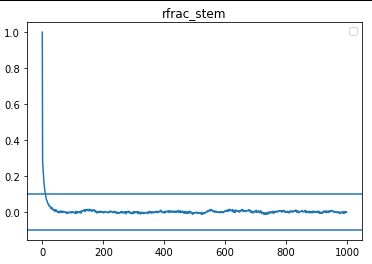 |
| 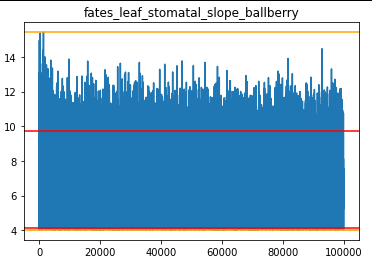 | 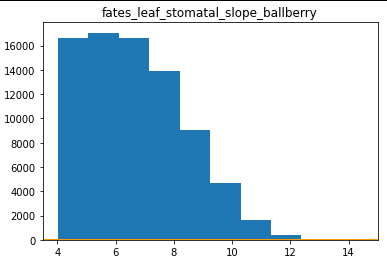 | 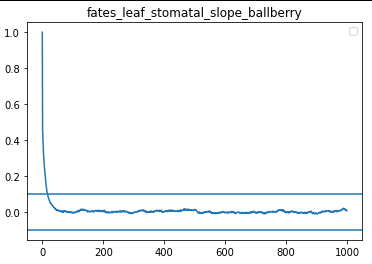 |
| 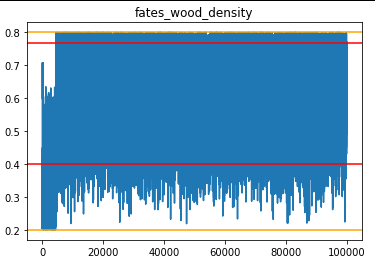 | 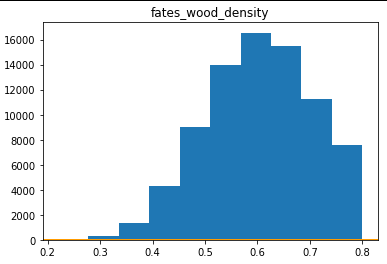 | 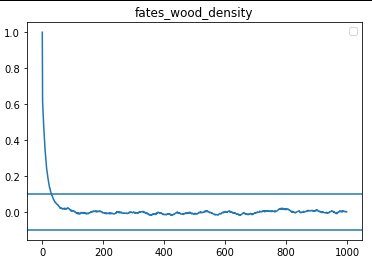 |
| 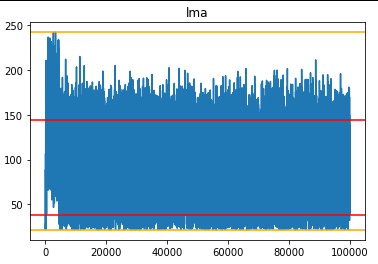 | 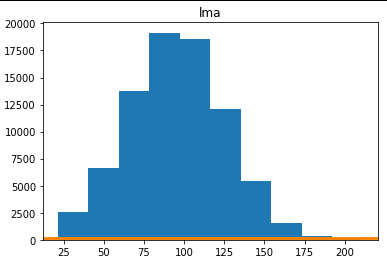 | 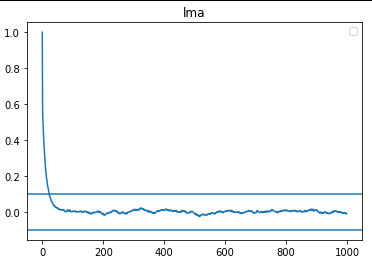 |
| 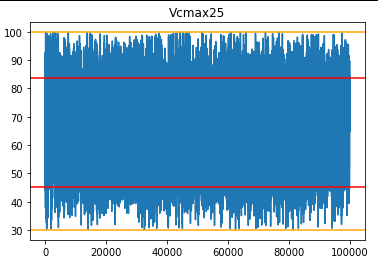 | 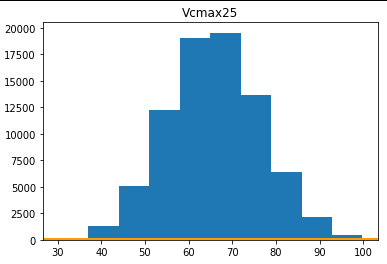 | 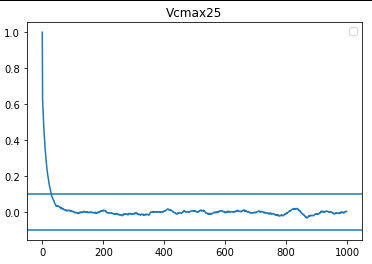 |


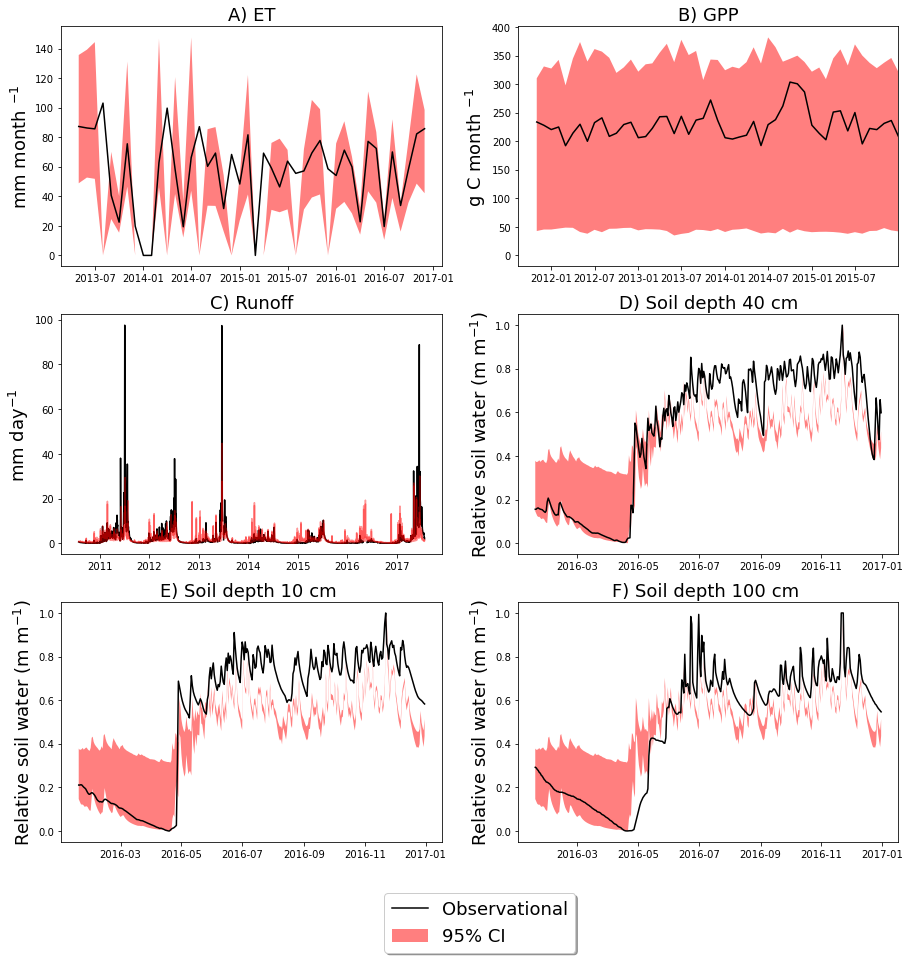


**Figure S.3**: Comparison of observations (black) and 54 FATES-HYDRO simulated trait assemblages for Barro Colorado Island (red) selected through a MCMC parameterization process. Trait assemblages are compared against observed (a) evapotranspiration (ET), (b) gross primary productivity (GPP), runoff and soil water content at three depths (10,40, 100 cm). 95% CI represents predictions from 95% of the models selected after MCMC burn-in process (10,000 samples).


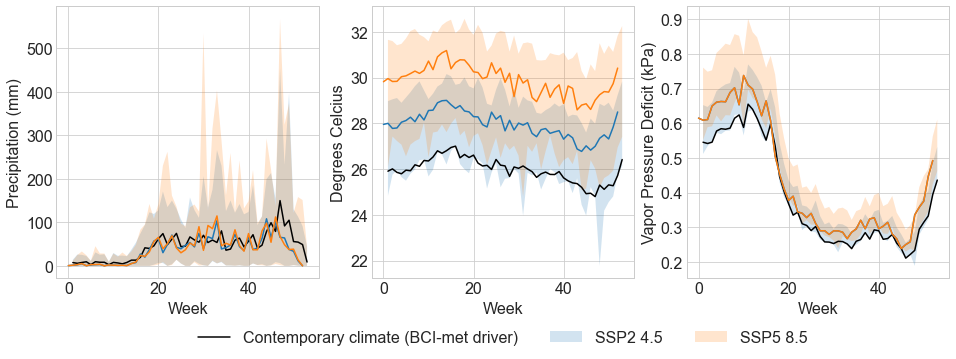


**Figure S.4:** Weekly ensemble means from the 16 climate models for SSP2-4.5 (blue) and SSP5-8.5 (orange) scenarios and Barro Colorado Island (BCI) met station data (contemporary climate) for precipitation, temperature and vapor pressure deficit used to drive the BCI FATES-HYDRO model. Ribbons represent the range of the models used in future simulations.

**Additional simulation results**


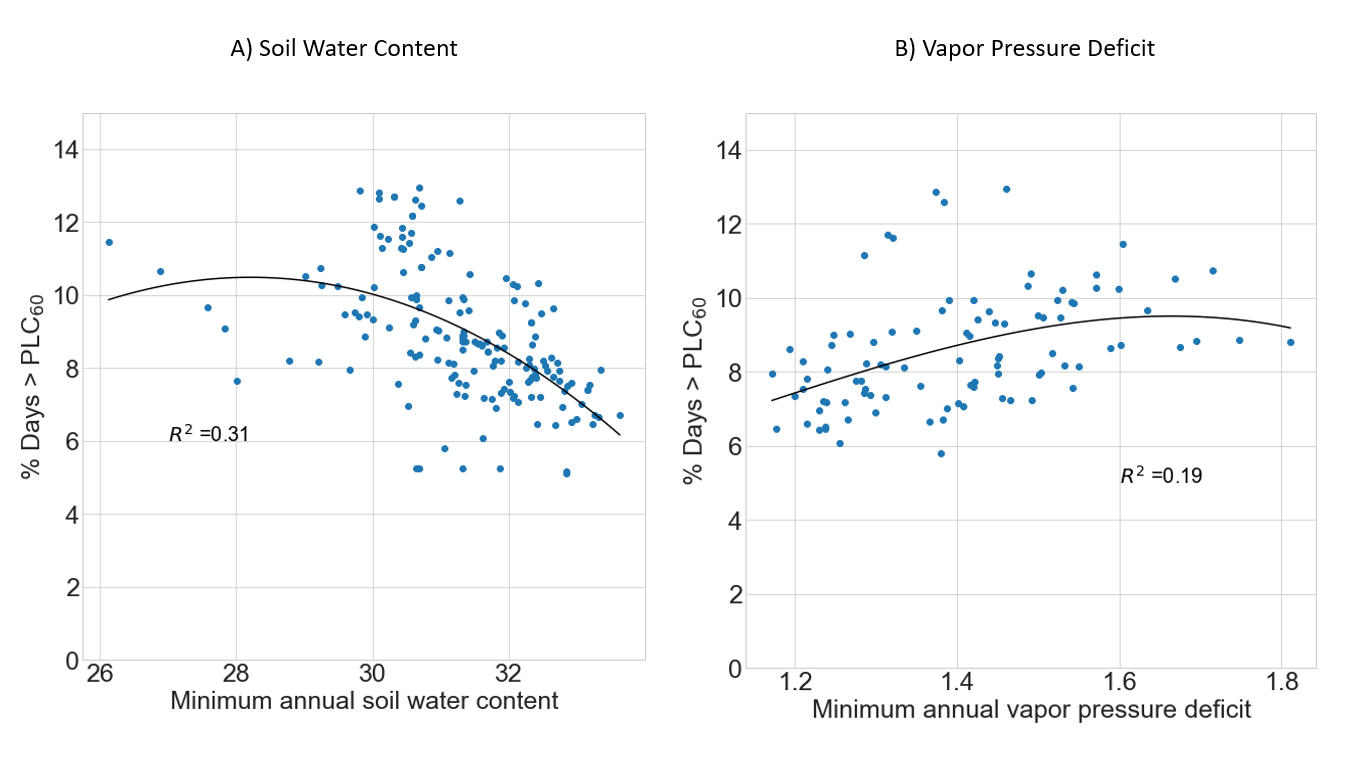


**Figure S.5:** Correlations between 60% percent loss of conductivity (PLC_60_) and minimum annual soil water content and vapor pressure deficit from future FATES-HYDRO simulations of the Barro Colorado Island, Panama, for emissions scenarios SSP-245, and SSP-585. Data points represent a single climate model for a single emissions scenario averaged across all tested plant trait assemblages.


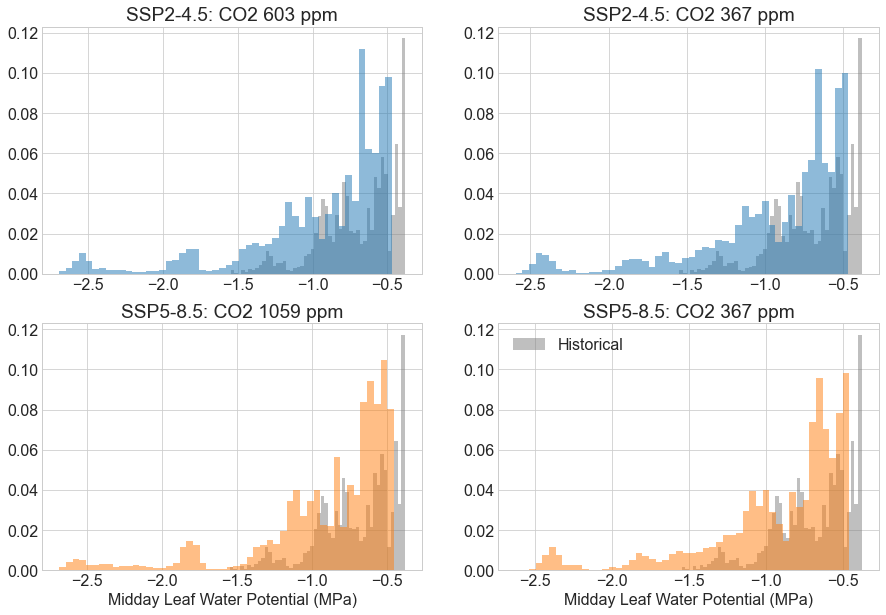


**Figure S.6:** Change in dry-season (mid-December- early May) minimum midday leaf water potential (MPa) simulated using the FATES-HYDRO model of Barro Colorado Island (BCI), Panama, for the two future climate scenarios (blue: SSP2-4.5 and orange: SSP5-8.5) with (left) and without (right) corresponding CO_2_ concentrations compared with the BCI historical climate driver (gray, 2010-2016). Minimum midday leaf water potential represents the point of greatest hydraulic strain on the plant.


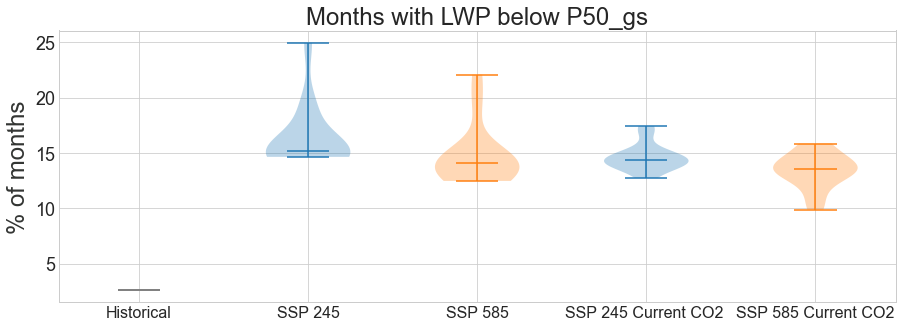

**Figure S.7:** Comparison of months with leaf water potential (LWP) below ψ50_gs_ , across climate scenarios projected by FATES-HYDRO model at Barro Colorado Island, Panama, under the historical climate driver, the two CMIP6 emissions scenarios (blue: SSP2-4.5 and orange: SSP5-8.5) and two corresponding CO_2_ levels.


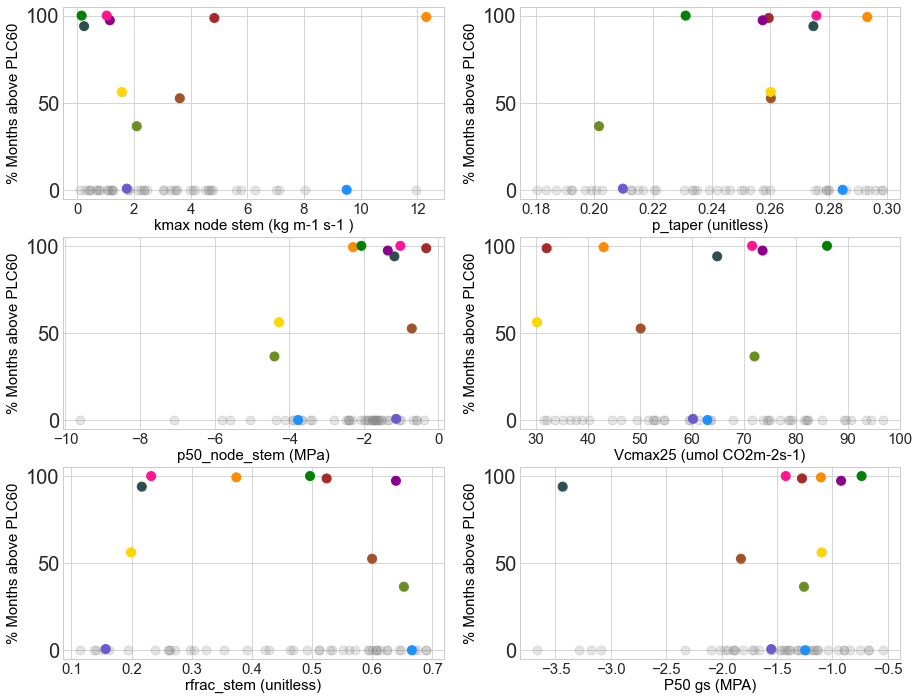


**Figure S.8:** Traits as a function of risk of hydraulic failure (% months with greater than 60 % loss of conductivity; PLC_60_) from FATES-HYDRO model of Barro Colorado Island outputs averaged across SSP2-4.5 and SSP5-8.5 scenarios. Colored dots are same plant trait assemblages across panels to allow for the tracking of multiple trait values, gray dots are those trait assemblages that did not experience any months with PLC_60_ greater than 0.0.


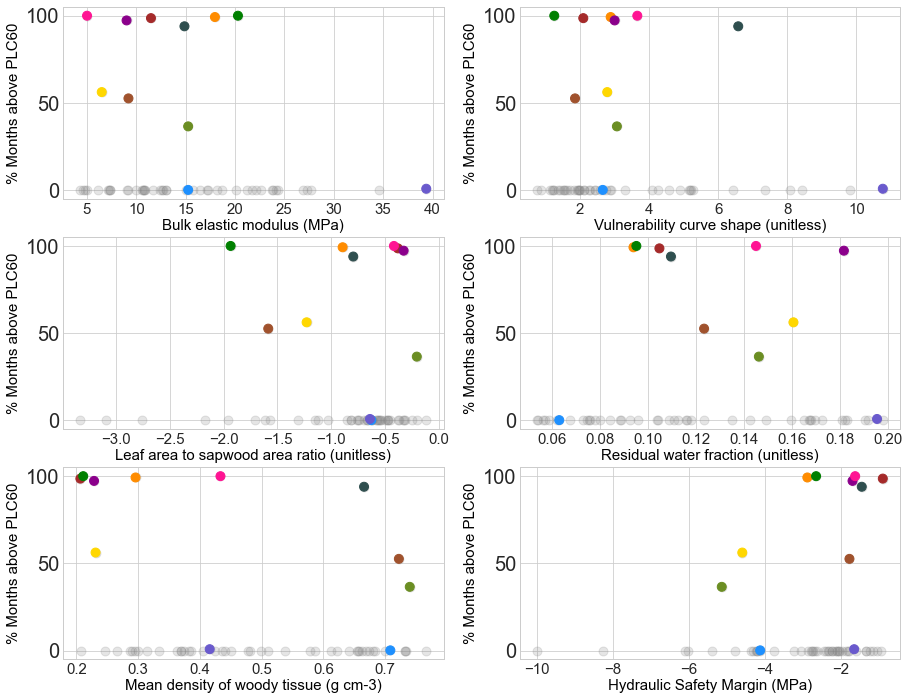


**Figure S.9**: Additional traits as a function of risk of hydraulic failure (% months with greater than 60 % loss of conductivity; PLC_60_) from FATES-HYDRO model of Barro Colorado Island outputs averaged across SSP2-4.5 and SSP5-8.5 scenarios. Colored dots are same plant trait assemblages across panels to allow for the tracking of multiple trait values, gray dots are those trait assemblages that did not experience any months withPLC_60_ greater than 0.0.

**Table S.4:** Statistically significant variables with correlation R^2^ >0.01 with PLC_60_ from future anomaly CMIP6 simulations of the FATES-HYDRO model for Barro Colorado Island.

| Variable | R^2^ |
| --- | --- |
| thetas_node_stem | 0.07 |
| avuln_node_troot | 0.04 |
| p_taper | 0.04 |
| roota_par | 0.03 |
| resid_node_stem | 0.02 |
| Specific_leaf\|_area | 0.01 |

**References**

Condit, R., Pérez, R., Lao, S., Aguilar, S., & Hubbell, S. P. (2017). Demographic trends and climate over 35 years in the Barro Colorado 50 ha plot. *Forest Ecosystems*, *4*(1), 17. <https://doi.org/10.1186/s40663-017-0103-1>

Koven, C. D., Knox, R. G., Fisher, R. A., Chambers, J. Q., Christoffersen, B. O., Davies, S. J., Detto, M., Dietze, M. C., Faybishenko, B., Holm, J., Huang, M., Kovenock, M., Kueppers, L. M., Lemieux, G., Massoud, E., McDowell, N. G., Muller-Landau, H. C., Needham, J. F., Norby, R. J., … Xu, C. (2020). Benchmarking and parameter sensitivity of physiological and vegetation dynamics using the Functionally Assembled Terrestrial Ecosystem Simulator (FATES) at Barro Colorado Island, Panama. *Biogeosciences*, *17*(11), 3017–3044. <https://doi.org/10.5194/bg-17-3017-2020>

Xu, C., Christoffersen, B., Robbins, Z., Knox, R., Fisher, R. A., Chitra-Tarak, R., Slot, M., Solander, K., Kueppers, L., Koven, C., & McDowell, N. (2023). Quantification of hydraulic trait control on plant hydrodynamics and risk of hydraulic failure within a demographic structured vegetation model in a tropical forest (FATES–HYDRO V1.0). *Geoscientific Model Development*, *16*(21), 6267–6283. <https://doi.org/10.5194/gmd-16-6267-2023>
